# Increase of EEG Spectral Theta Power Indicates Higher Risk of the Development of Severe Cognitive Decline in Parkinson’s Disease after 3 Years

**DOI:** 10.3389/fnagi.2016.00284

**Published:** 2016-11-29

**Authors:** Vitalii V. Cozac, Menorca Chaturvedi, Florian Hatz, Antonia Meyer, Peter Fuhr, Ute Gschwandtner

**Affiliations:** ^1^Department of Neurology, Hospital of the University of BaselBasel, Switzerland; ^2^Department of Mathematics and Computer Science, University of BaselBasel, Switzerland

**Keywords:** EEG, cognitive tests, Parkinson’s disease, cognitive decline, cohort study

## Abstract

**Objective:** We investigated quantitative electroencephalography (qEEG) and clinical parameters as potential risk factors of severe cognitive decline in Parkinson’s disease.

**Methods:** We prospectively investigated 37 patients with Parkinson’s disease at baseline and follow-up (after 3 years). Patients had no severe cognitive impairment at baseline. We used a summary score of cognitive tests as the outcome at follow-up. At baseline we assessed motor, cognitive, and psychiatric factors; qEEG variables [global relative median power (GRMP) spectra] were obtained by a fully automated processing of high-resolution EEG (256-channels). We used linear regression models with calculation of the explained variance to evaluate the relation of baseline parameters with cognitive deterioration.

**Results:** The following baseline parameters significantly predicted severe cognitive decline: GRMP theta (4–8 Hz), cognitive task performance in executive functions and working memory.

**Conclusions:** Combination of neurocognitive tests and qEEG improves identification of patients with higher risk of cognitive decline in PD.

## Introduction

The progression of Parkinson’s disease (PD) is associated with cognitive decline and dementia (Aarsland et al., 2005; Goldman et al., 2012). Dementia in PD reaches about 30% of all cases with PD (Emre, 2003). The risk of dementia is about 80% for the patients living for more than 20 years with PD (Hely et al., 2008). Early and correct identification of the patients with the risk of severe cognitive decline is a challenging problem of neurology, which has led to the suggestion of various markers of cognitive decline in PD (Mollenhauer et al., 2014). Quantitative electroencephalography (qEEG) – digital processing of EEG recordings to obtain numerical and graphical data – showed that the power (the square of amplitudes of electrical activity) of the brain in PD patients with cognitive impairment is increased in the frequency range below 8 Hz, and decreased in the range above 8 Hz (Caviness et al., 2007; Fonseca et al., 2009; Babiloni et al., 2011). Additionally, in cohort studies qEEG produced promising results in predicting progression to dementia in PD (Bonanni et al., 2008; Klassen et al., 2011; Gu et al., 2014; Olde Dubbelink et al., 2014).

The purpose of our study was to investigate clinical and qEEG parameters as predictors of severe cognitive decline in PD, using high-resolution EEG with 256 electrodes and with fully automated removal of artifacts (Hatz et al., 2015). Our hypothesis was that qEEG variables at baseline are able to predict severe cognitive decline, and these qEEG variables are not influenced by clinical and demographic parameters. To address this research question a prospective (3 years) cohort of PD patients was assessed for potential neurological, psychological, and neurophysiological risk factors.

## Materials and Methods

### Enrollment of the Patients

Patients were recruited from the outpatient clinic of the Department of Neurology and Neurophysiology of the Hospital of the University of Basel (City of Basel, Switzerland) in the period from 2011 to 2012. Selection criteria: PD according to United Kingdom Parkinson’s Disease Society Brain Bank criteria (Gibb and Lees, 1988). The patients who had dementia (Diagnostic and Statistical Manual of Mental Disorders, 4th Edition), history of stroke, epilepsy, multiple sclerosis and surgical interventions to the brain, or/and insufficient knowledge of German language, were excluded. Included patients underwent neurological, cognitive and qEEG examinations on inclusion (baseline) and after a mean interval of 3 years (follow-up). Specialists who performed the assessment of the patients (neurologists, neuropsychologists, and technicians) were unaware of the details of this study.

### Standard Protocol Approvals, Registrations, and Patient Consents

The research ethics committee of the Cantons of Basel (*Ethikkommission beider Basel*) approved this study. All patients were fully informed of the nature of the study and provided written consent to participate.

### Neurological Assessment

Subsection III (motor examination) of the Unified Parkinson’s Disease Rating Scale (UPDRS-III) was filled out. Levodopa equivalent of the daily dose of the antiparkinsonian medication was calculated (LEDD, Tomlinson et al., 2010). Disease duration was assessed since the first symptoms of PD reported by the patient or caregiver.

### Cognitive Assessment

Cognitive evaluation was performed in individual sessions divided in three parts; each part with duration of approximately 90 min per day. The interval between the parts of each session was between 24 and 48 h. Mini-Mental State Examination and a battery of 14 cognitive tests were applied. Test variables were normalized with reference to a normative data base of 604 healthy controls from the Memory Clinic, Felix Platter Hospital of Basel, Switzerland (Berres et al., 2000). Cognitive tests were grouped in six cognitive domains (Zimmermann et al., 2015): “attention,” “executive functions,” “fluency,” “long-term memory,” “working memory,” and “visual-spatial functions” (**Table 1**). A score reflecting cognitive performance in each domain comprised mean of the constituent test variables. Additionally, an overall cognitive score comprised a mean of all 14 cognitive tests. Mood and behavior were assessed with tests: Beck Depression Inventory-II, and compartment “Emotional well-being” (six items with five-step gradation) of the Parkinson’s disease Questionnaire with 39 items.

**Table 1:** Cognitive tests and cognitive domains.

Domain	Tests within a domain
(1) Attention	• Stroop Color-Word: time for color naming
	• Trail-Making: time for part A
	• Digit Span: correct backward
(2) Executive functions	• Trail-Making: time for part B divided by time for part A
	• Stroop Color-Word: time for interference task divided by time for color naming
	• Wisconsin Card Sorting: number of errors
(3) Fluency	• Phonemic verbal fluency: correct answers
	• Semantic verbal fluency: correct answers
(4) Long-term memory	• Verbal Learning – long-delayed recall
	• Verbal Learning – discrimination
(5) Working memory	• Corsi blocks: correct forward
	• Divided attention: omissions
(6) Visual-spatial functions	• Block design
	• Rey-Osterrieth complex figure copy


### Neurophysiological Assessment

**FIGURE 1:**
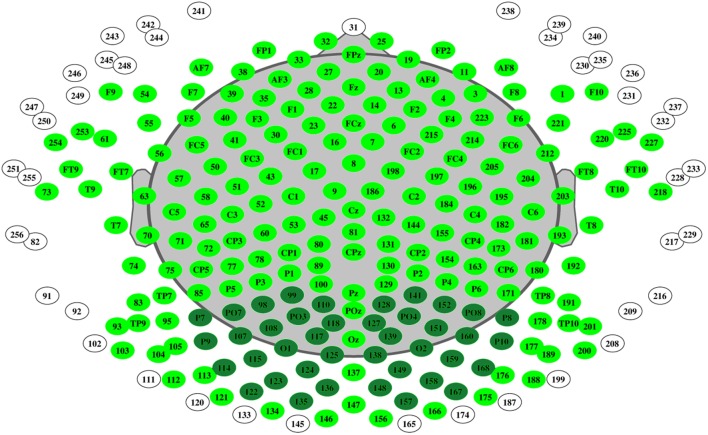
**Electrode mapping of 256 electrodes.** Active electrodes colored in dark and light green, occipital electrodes – dark green.

Continuous EEG with 256 electrodes (214 active electrodes) was recorded in relaxed eyes-closed state of the patients (Net Station 300; Electrical Geodesics, Inc). Electrode located at *C*_Z_ was used as reference. The sampling rate was set at 1000 Hz, oscillations were filtered with 2500 order least-square filter with band-pass 0.5–70 Hz, and notch 50 Hz. Spectral analysis was performed with “TAPEEG” toolbox (Hatz et al., 2015) by multitaper method (Percival and Walden, 1993). Detection and removal of artifacts (e.g., eye blinks) was fully automated, by an independent component analysis. Channels with bad activations were automatically detected and interpolated by spherical spline method. Global relative median power (GRMP) was calculated in frequency ranges: delta (1–4 Hz), theta (4–8 Hz), alpha1 (8–10 Hz), alpha2 (10–13 Hz), and beta (13–30 Hz). Additionally, median value in the frequency range 4–14 Hz was calculated as the 50% quantile of the overall power spectrum from occipital electrodes – occipital median frequency (**Figure 1**).

### Statistics

Statistical calculations were performed with R tool for statistical calculations (R Core Team, 2015). The normality of the distribution of the data was tested with Shapiro–Wilk test. The influence of the baseline parameters on cognitive state at follow-up was checked with univariate and multivariate linear regression models with backward elimination. Prediction accuracy was checked with receiver operating characteristic (ROC) curves. The results were additionally checked with Random Forest method with regression. The level of statistical significance was set at 0.05.

#### Cognitive Outcome

A change index in overall cognitive score (CI-OCS) was used as outcome. The CI-OCS was calculated as difference in overall cognitive score between follow-up and baseline, divided by the standard error of the difference (Jacobson and Truax, 1991).

#### Regression Models

The following baseline variables were considered as predictors: GRMP in ranges delta, theta, alpha1, alpha2, and beta, MF, cognitive domains: “attention,” “executive functions,” “fluency,” “long-term memory,” “working memory,” and “visual-spatial functions,” age, sex, highest educational level (measured in years), disease duration (years), duration of observation (years), LEDD, and UPDRS-III. Significant variables from the univariate regression models were included in multivariate models. To check the added value of the significant predictors to the cognitive task performance, non-normalized to 100% explained variance of the models was calculated. The relative importance of the variables was calculated with the R package “relaimpo” (Grömping, 2006) with method “LMG” and plotted in a bar diagram.

#### ROC-Curves

The ROC-curve analyses were performed with the R package “pROC” (Robin et al., 2011). We categorized the sample on the basis of MMSE score at follow-up (cut off <24).

#### Random Forest with Regression

Random Forest (Hastie et al., 2009) is an ensemble machine learning method used for classifying data with high accuracy and for regression analysis. The goal of this method is to reduce the variance in the data and get a higher predictive performance of the model. This is done by using several decision trees, which are constructed based on subsets of the same training data, and then getting predictions on the test set based on the training. Each variable included in the model is evaluated based on its effect on the overall accuracy of the model and is ranked higher up if its exclusion results in a drop in the model accuracy. The role of each variable in the classification process is reflected in output measures called mean decrease accuracy (MDA) and mean decrease gini coefficient (MDGC). MDA is the increase in mean squared error of predictions after predictor variables being randomly shuffled. Higher the MDA, the more important is the variable. MDGC relates to the decrease of node impurity in the decision tree after each split, summed over all splits and trees. Higher the MDGC is, the more important the variable. Random Forest analysis was performed with the R package “randomForest” (Liaw and Wiener, 2002).

## Results

### Enrollment of the Patients

Between January 2012 and December 2013, 55 patients were selected in the study and assessed for neurological, psychological and qEEG parameters (Supplement 1). At follow-up, cognitive outcome along with the other clinical data was obtained for 37 patients. Thus, these 37 patients were included in the analysis (**Table 2**). Changes in neurological and cognitive features of the sample are shown in Supplement 2.

**Table 2:** Sample at baseline.

Factors	Values
Sex, males/females	25/12
Age, years	67 [31, 84]
Disease duration, years	8 [1, 20]
Duration of observation, months	37 [30, 44]
Education, years	14 [9, 20]
Beck Depression Inventory-II	6 [0, 15]
Obsessive Compulsive Inventory	6 [0, 25]
PDQ39 – emotional well-being	17 [0, 50]
UPDRS, Subscale III	14 [0, 50]
Levodopa equivalent, mg per day	691 [150, 2129]
Attention	-0.02 [-2.07, 1.13]
Executive functions	-0.03 [-3.73, 1.17]
Fluency	-0.18 [-1.93, 1.61]
Long-term memory	-0.13 [-1.62, 2.60]
Working memory	-0.23 [-1.50, 2.37]
Visual-spatial functions	-0.23 [-2.60, 1.79]
Overall cognitive score	-0.10 [-2.05, 0.96]
Mini Mental State	29 [24, 30]
GRMP delta, %	22 [9, 42]
GRMP theta, %	18 [10, 46]
GRMP alpha1, %	18 [5, 33]
GRMP alpha2, %	13 [5, 33]
GRMP beta, %	20 [10, 38]
Median frequency, Hz	8.71 [7.14, 9.99]


*For continuous variables presented as median and range (*M* [min, max]). GRMP, global relative median power; PDQ39, Parkinson’s disease questionnaire with 39 items; UPDRS, unified Parkinson’s disease rating scale.*

### Influence on CI-OCS

Regression analyses (Supplement 3, Tables 3–6) identified three baseline parameters which had significant influence on CI-OCS: GRMP theta (β = -3.16, *p* < 0.001), cognitive domain “executive functions” (β = 0.54, *p* < 0.001), cognitive domain “working memory” (β = 0.19, *p* < 0.05), adjusted *R* squared = 0.64, *p* < 0.001. Explained variance of the overall model was 66.9%, of which “executive functions” made 27.5%, GRMP theta – 25.8%, and “working memory” – 13.6% (Supplement 3, Table 6).

**FIGURE 2:**
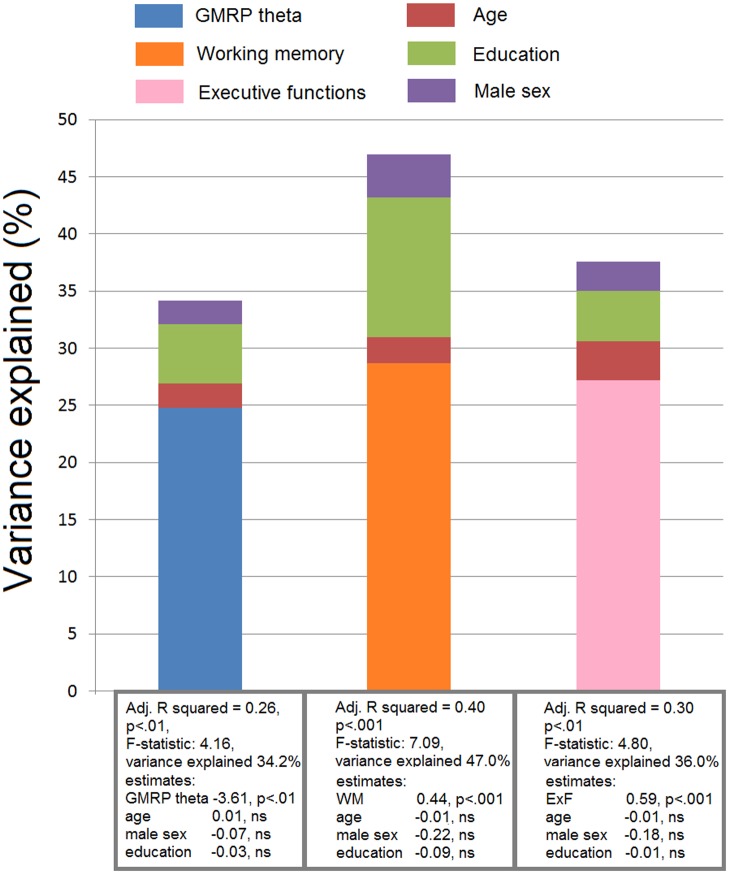
**Results of the linear regression analyses.** Confounding effect of age, male sex, and education on the significant predictors of cognitive decline (GMRP theta, executive functions, and working memory). The variance of the models, that is explained by these predictors, is shown.

Additionally, we checked if age, sex, and education had confounding effect on each of the three significant variables (GRMP theta, “executive functions,” and “working memory”). No confounding effects were identified (**Figure 2**).

### ROC-Curve Analyses

**FIGURE 3:**
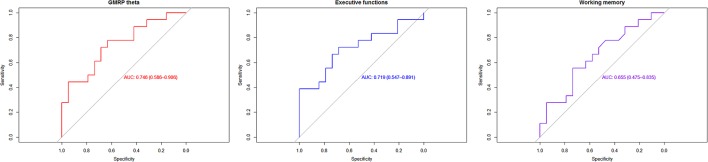
**Receiver operating characteristic (ROC)-curves analyses**.

Receiver operating characteristic were built using variables: GRMP theta, “executive functions,” and “working memory.” Best accuracy was identified in GRMP theta: AUC = 75%, specificity = 63%, specificity = 77% (**Figure 3**; Supplement 3, Table 7).

### Random Forest

Global relative median power theta was classified as the most important variable (MDA = 7.49, MDGC = 1.63) (Supplement 3, Table 8).

## Discussion

In our observation, increase of GRMP theta (4–8 Hz), and decrease of cognitive performance in domains “executive functions” and “working memory” significantly predicted worse CI-OCS after 3 years.

Our findings in GRMP theta are in line with the data from cohort studies with semi-automated processing of EEG (Klassen et al., 2011; Olde Dubbelink et al., 2014). Additionally, in cross-sectional comparisons between Parkinson’s disease patients with dementia and matched healthy controls, spectral power in the frequency range below 8 Hz was significantly increased in demented patients (Babiloni et al., 2011; Fonseca et al., 2013). From a pathophysiologic perspective, EEG slowing in severe cognitive decline may be explained by disruption of thalamo-cortical circuits, and pathological synchronization of the brain motor systems with slow frequencies related to the sensory motor integration (Steriade et al., 1990; Rossini et al., 1991). We can speculate that these pathological changes precede clinical manifestation of cognitive decline in PD.

With regard to cognitive factors, we found that worse scores in domains “executive functions” and “working memory” are significant predictors of cognitive decline. Zimmermann et al. (2015) in a cross-sectional analysis showed significant correlation of occipital median frequency with overall cognitive score, domains “executive functions,” “long-term memory,” “attention,” and “fluency” in dementia-free patients with PD. Olde Dubbelink et al. (2014) found that fronto-executive (spatial span score) and posterior (pattern recognition memory) significantly predicted dementia in Parkinson’s disease. Impairment of the executive functions is common in the early stage of PD (Kehagia et al., 2010). However, the cognitive profile of early stage PD is heterogeneous, and the significance of domain-specific cognitive deficits in identifying patients with a risk of dementia is still studied (Robbins and Cools, 2014).

Our findings demonstrate that a combination of neurocognitive tests with qEEG improves identification of patients with PD and higher risk of cognitive decline. While it is important for practical reasons to identify strong risk factors for dementia developing within 1 or 2 years, the short mean observation period of 3 years is a limitation of the study, and a longer follow-up is warranted. Another limitation is the relatively small sample size. Strengths of the study include the comprehensive neuropsychological and psychiatric assessments as well as the fully automated processing of high-resolution EEG, which could be implemented in clinical practice as a universally available technique. Additionally, automated processing of EEG facilitates the application of advanced analyses of the cognitive function.

## Conclusion

High GRMP theta, especially when combined with poorer cognitive scores in “executive functions” and “working memory,” identifies patients with PD who are at a higher risk of progression to dementia.

## Author Contributions

VC study concept and design, acquisition of data, writing of the first draft. MC analysis and interpretation of data. FH analysis and interpretation of data, critical revision of manuscript for intellectual content. AM analysis and interpretation of data, critical revision of manuscript for intellectual content. PF analysis and interpretation of data, study supervision, critical revision of manuscript for intellectual content. UG acquisition of data, analysis and interpretation of data, study supervision, critical revision of manuscript for intellectual content.

## Conflict of Interest Statement

The authors declare that the research was conducted in the absence of any commercial or financial relationships that could be construed as a potential conflict of interest.
