# Supplementary material for: Increase of EEG Spectral Theta Power Indicates Higher Risk of the Development of Severe Cognitive Decline in Parkinson’s Disease after 3 Years
Source: Front Aging Neurosci. 2016 Nov 29;8:284. doi: 10.3389/fnagi.2016.00284 (PMC5126063; doi:10.3389/fnagi.2016.00284)
Supplement: Supplementary file 1 [file Data_Sheet_1.DOCX]

**Supplement 1. Flow chart of the study.**

Patients with parkinsonism screened in the out-patient clinic of the Hospital oft he University of Basel
**n=197**

Agreed to participate in the study and investigated at baseline
**n=55**

Investigated at follow-up **n=37**

Patients with Parkinson’s disease who fit to the criteria of the study
**n=103**

Drop out from the study **n=18**:
- lost contact n=8
- refused to continue after DBS n=3
- refused with unknown reason n=2
- severe health problem (other than PD) n=2
- refused due to change of residence n=2
- death n=1
