# Supplementary material for: Increase of EEG Spectral Theta Power Indicates Higher Risk of the Development of Severe Cognitive Decline in Parkinson’s Disease after 3 Years
Source: Front Aging Neurosci. 2016 Nov 29;8:284. doi: 10.3389/fnagi.2016.00284 (PMC5126063; doi:10.3389/fnagi.2016.00284)
Supplement: Supplementary file 2 [file Data_Sheet_2.DOCX]

Supplement 2. Frontiers in Aging Neuroscience. Cozac, Chaturvedi, Hatz, Meyer, Fuhr, Gschwandtner.

**Fig. 4. Cognitive domains and overall cognitive score of the sample at baseline and after 3 years.**

**
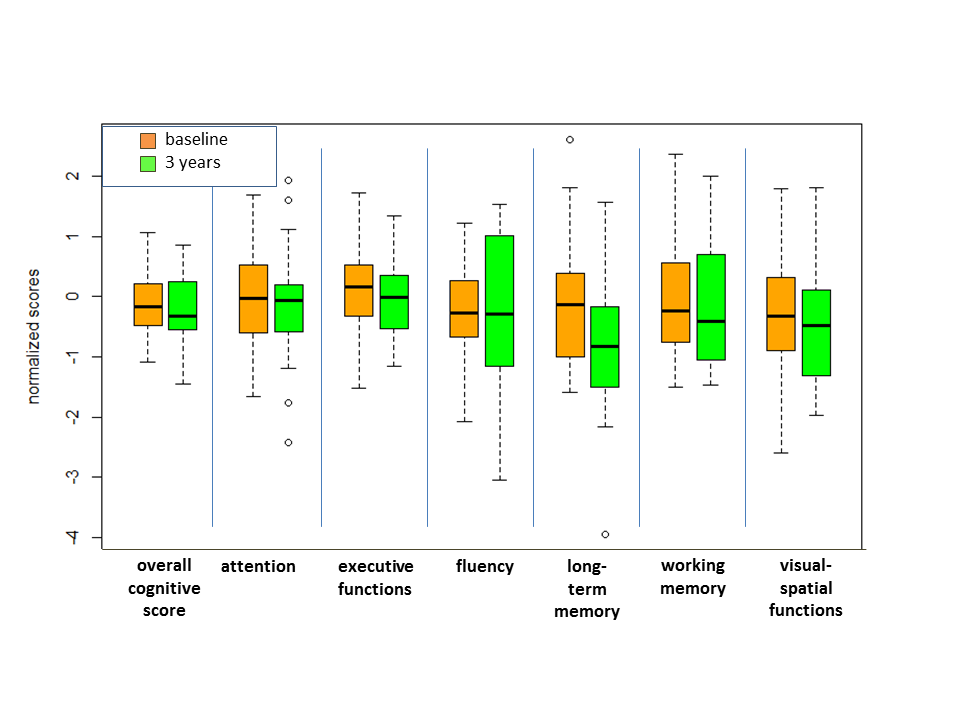
**

**Fig. 5. Clinical parameters of the sample at baseline and after 3 years.
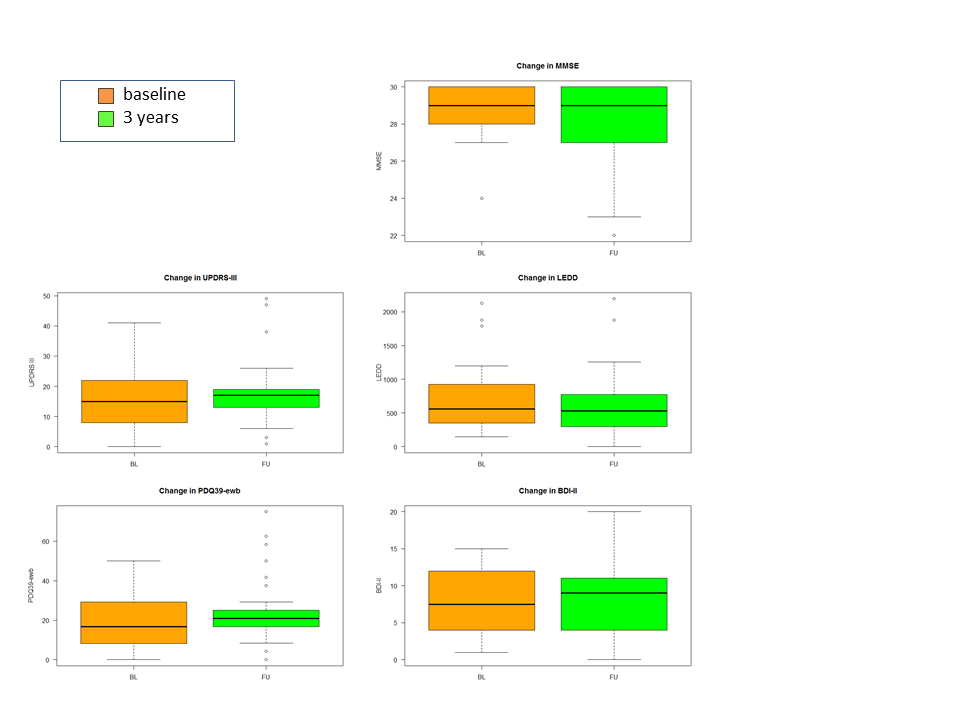
**
