# Supplementary material for: Increase of EEG Spectral Theta Power Indicates Higher Risk of the Development of Severe Cognitive Decline in Parkinson’s Disease after 3 Years
Source: Front Aging Neurosci. 2016 Nov 29;8:284. doi: 10.3389/fnagi.2016.00284 (PMC5126063; doi:10.3389/fnagi.2016.00284)
Supplement: Supplementary file 3 [file Data_Sheet_3.DOCX]

Supplement 3. Frontiers in Aging Neuroscience. Cozac, Chaturvedi, Hatz, Meyer, Fuhr, Gschwandtner.

**Table 3. Univariate regression models. In all models “Change index of the overall cognitive score” was introduced as dependent variable.**

| Predictor | Estimate | Standard error | Adj. R-squared | F-statistic | t value | p-value |
| --- | --- | --- | --- | --- | --- | --- |
| age | -0.020 | 0.013 | 0.075 | 3.897 | -1.974 | 0.0563 |
| male.sex | -0.360 | 0.174 | 0.083 | 4.256 | -2.063 | 0.0505 |
| education | -0.008 | 0.024 | 0.117 | 3.440 | -0.307 | 0.0519 |
| duration.observation | 0.037 | 0.286 | -0.028 | 0.016 | 0.130 | 0.2575 |
| disease.duration | -0.032 | 0.018 | 0.055 | 3.131 | -1.770 | 0.0855 |
| updrs.iii.baseline | -0.017 | 0.009 | 0.067 | 3.590 | -1.895 | 0.0664 |
| ledd.baseline | -0.000 | 0.000 | -0.008 | 0.681 | -0.826 | 0.4146 |
| nms.baseline | -0.002 | 0.004 | -0.019 | 0.328 | -0.573 | 0.5704 |
| bdi.ii.baseline | -0.020 | 0.020 | -0.001 | 0.930 | -0.965 | 0.3414 |
| pdq39.ewb.baseline | 0.000 | 0.006 | -0.028 | 0.004 | 0.064 | 0.9495 |
| mmse.baseline | 0.030 | 0.073 | -0.023 | 0.169 | 0.411 | 0.6834 |
| attention.baseline | 0.332 | 0.129 | 0.135 | 6.617 | 2.572 | 0.0145* |
| exec.functions.baseline | 0.560 | 0.137 | 0.027 | 14.300 | 3.782 | 0.0005* |
| fluency.baseline | 0.381 | 0.134 | 0.164 | 8.070 | 2.841 | 0.0007* |
| long.memory.baseline | 0.250 | 0.118 | 0.088 | 4.476 | 2.116 | 0.0415* |
| work.memory.baseline | 0.3177 | 0.104 | 0.185 | 9.195 | 3.032 | 0.0045* |
| vis.spat.funct.baseline | 0.3122 | 0.106 | 0.173 | 8.543 | 2.923 | 0.0060* |
| delta.baseline | -0.379 | 1.413 | -0.026 | 0.072 | -0.268 | 0.7900 |
| theta.baseline | -3.289 | 0.900 | 0.255 | 13.360 | -3.655 | 0.0008* |
| alpha1.baseline | 1.434 | 1.296 | 0.006 | 1.225 | 1.107 | 0.2759 |
| alpha2.baseline | 3.247 | 1.650 | 0.073 | 3.871 | 1.968 | 0.0470* |
| beta.baseline | 3.364 | 1.553 | 0.093 | 4.692 | 2.166 | 0.0372* |
| median.freq.baseline | 0.387 | 0.148 | 0.138 | 6.791 | 2.606 | 0.0133* |

**Table 4.1. Multivariate regression model with significant cognitive predictors (domains: attention, executive functions, and fluency), selected in univariate models. “Change index of the overall cognitive score” was introduced as dependent variable.**

| Predictor | Estimate | Standard error | t value | p-value | Variance importance metrics, % |
| --- | --- | --- | --- | --- | --- |
| attention.baseline | 0.129 | 0.138 | 0.932 | 0.3583 | 7.36 |
| exec.functions.baseline | 0.427 | 0.159 | 2.684 | 0.0113* | 20.01 |
| fluency.baseline | 0.167 | 0.149 | 1.119 | 0.271 | 9.15 |
| Residual standard error: 0.5622, F-statistic: 6.332 on 3 and 33 DF, Adjusted R-squared: 0.3033, p-value: 0.001638  Proportion of variance explained by model: 36.52%, metrics are not normalized. | | | | | |

**Table 4.2. Multivariate regression model with significant cognitive predictors (domains: long-term memory, working memory, and visual-spatial functions), selected in univariate models . “Change index of the overall cognitive score” was introduced as dependent variable.**

| Predictor | Estimate | Standard error | t value | p-value | Variance importance metrics, % |
| --- | --- | --- | --- | --- | --- |
| long.memory.baseline | 0.150 | 0.110 | 1.357 | 0.1840 | 6.96 |
| work.memory.baseline | 0.236 | 0.103 | 2.276 | 0.0295* | 15.01 |
| vis.spat.funct.baseline | 0.193 | 0.108 | 1.778 | 0.0845 | 12.41 |
| Residual standard error: 0.5715, F-statistic: 5.768 on 3 and 33 DF, Adjusted R-squared: 0.2844, p-value: 0.002755  Proportion of variance explained by model: 34.38%, metrics are not normalized. | | | | | |

**Table 5. Multivariate regression model with significant qEEG spectral predictors, selected in univariate models . “Change index of the overall cognitive score” was introduced as dependent variable.**

| Predictor | Estimate | Standard error | t value | p-value | Variance importance metrics, % |
| --- | --- | --- | --- | --- | --- |
| theta.baseline | -5.267 | 2.084 | -2.527 | 0.0167* | 17.67 |
| alpha2.baseline | -4.4759 | 3.976 | -1.126 | 0.2687 | 4.16 |
| beta.baseline | -2.034 | 2.398 | -0.848 | 0.4026 | 4.05 |
| med.freq.baseline | 0.195 | 0.435 | 0.449 | 0.6568 | 5.64 |
| Residual standard error: 0.5928, F-statistic: 3.688 on 4 and 32 DF, Adjusted R-squared: 0.2300, p-value: 0.01404  Proportion of variance explained by model: 31.52%, metrics are not normalized. | | | | | |

**Table 6. Multivariate regression model with significant qEEG spectral and cognitive predictors. “Change index of the overall cognitive score” was introduced as dependent variable.**

| Predictor | Estimate | Standard error | t value | p-value | Variance importance metrics, % |
| --- | --- | --- | --- | --- | --- |
| theta.baseline | -3.157 | 0.641 | -4.920 | 2.33e-05 * | 25.79 |
| exec.functions.baseline | 0.544 | 0.106 | 5.127 | 1.27e-05 * | 27.52 |
| work.memory.baseline | 0.187 | 0.072 | 2.588 | 0.0142 * | 13.61 |
| Residual standard error: 0.4057, F-statistic: 22.280 on 3 and 33 DF, Adjusted R-squared: 0.6394, p-value: 4.542e-08  Proportion of variance explained by model: 66.92%, metrics are not normalized. | | | | | |

**Table 7. ROC-curves.**

| Coordinates | GMRP theta | Executive  functions | Working  memory |
| --- | --- | --- | --- |
| Area under the curve | 0.746 | 0.719 | 0.655 |
| Specificity | 0.631 | 0.684 | 0.736 |
| Sensitivity | 0.777 | 0.722 | 0.555 |
| Positive predictive value | 0.666 | 0.684 | 0.666 |
| Negative predictive value | 0.750 | 0.722 | 0.636 |

**Table 8. Random Forest analysis.**

| Predictors | Mean Decrease Accuracy | Mean Decrease Gini Coefficient |
| --- | --- | --- |
| theta.baseline | 7.49 | 1.63 |
| alpha2.baseline | 4.28 | 1.20 |
| beta.baseline | 4.98 | 1.29 |
| median.freq.baseline | 1.79 | 1.27 |
| attention.baseline | 2.91 | 1.40 |
| exec.functions.baseline | 7.29 | 1.67 |
| fluency.baseline | 4.39 | 1.51 |
| work.memory.baseline | 3.69 | 1.51 |
| long.memory.baseline | 1.38 | 1.25 |
| vis.spat.funct.baseline | 4.58 | 1.43 |
| Type of random forest: regression  Number of trees: 1000  No. of variables tried at each split: 1  Mean of squared residuals: 0.2796442, % Var explained: 37.03 | | |
